# Ultrashort Pulsed Laser Drilling of Printed Circuit Board Materials

**DOI:** 10.3390/ma15113932

**Published:** 2022-05-31

**Authors:** Daniel Franz, Tom Häfner, Tim Kunz, Gian-Luca Roth, Stefan Rung, Cemal Esen, Ralf Hellmann

**Affiliations:** 1 Applied Laser and Photonics Group, University of Applied Sciences Aschaffenburg, Würzburger Straße 45, 63743 Aschaffenburg, Germany; gian-luca.roth@th-ab.de (G.-L.R.); stefan.rung@th-ab.de (S.R.); ralf.hellmann@th-ab.de (R.H.); 2 Schmoll Maschinen GmbH, Odenwaldstraße 67, 63322 Rödermark, Germany; haefner@schmoll-maschinen.de (T.H.); tim.kunz@schmoll-maschinen.de (T.K.); 3 Applied Laser Technologies, Ruhr University Bochum, Universitätsstraße 150, 44801 Bochum, Germany; esen@lat.rub.de

**Keywords:** laser drilling, ultrashort pulse (USP) laser, printed circuit board (PCB), heat accumulation, pulse to pulse interactions, microvia, ablation threshold, materialography

## Abstract

We report on a comprehensive study of laser percussion microvia drilling of FR-4 printed circuit board material using ultrashort pulse lasers with emission in the green spectral region. Laser pulse durations in the pico- and femtosecond regime, laser pulse repetition rates up to 400 kHz and laser fluences up to 11.5 J/cm${}^{2}$ are applied to optimize the quality of microvias, as being evaluated by the generated taper, the extension of glass fiber protrusions and damage of inner lying copper layers using materialography. The results are discussed in terms of the ablation threshold for FR-4 and copper, heat accumulation and pulse shielding effects as a result of pulse to pulse interactions. As a specific result, using a laser pulse duration of 2 ps appears beneficial, resulting in small glass fiber protrusions and high precision in the stopping process at inner copper layer. If laser pulse repetition rates larger than 100 kHz are applied, we find that the processing quality can be increased by heat accumulation effects.

## 1. Introduction

A printed circuit board (PCB) is an electronic key component that requires the production of thousands vias with diameters ranging from 1 to 1000 µm [1,2]. Driven by the trend towards miniaturized high-performance electronic devices and power electronics, the electronic industry is challenged in minimizing via diameters, in turn enabling increasing wiring and interconnection densities. Mechanically produced through holes in multi-layer PCBs are an unfavorable solution because of the limited available diameter range and throughput. Thus, laser blind via drilling to inner copper layers has been established with laser beam sources such as CO${}_{2}$ or pulsed UV nanosecond lasers [3,4,5]. These so-called microvias enable the connection of several electrically conductive copper layers of a PCB by electroplating with copper. For the separation of the copper layers, layers of FR-4 laminate (composite of epoxy resin with woven fiberglass reinforcement [6]) are widely used in the assembly fabrication of PCBs.

**Figure 1:**
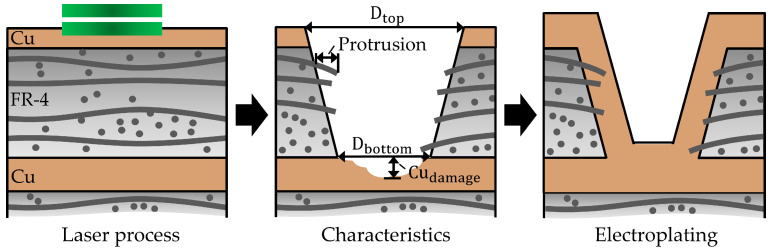
Laser-based fabrication of microvias in FR-4 PCB material and their technical specification based on the taper (ratio of the upper to lower diameter), glass fiber protrusion, and damage in the inner copper layer Cu${}_{\mathrm{damage}}$.

Characterization of microvias in typical FR-4 PCB material is based on the taper (ratio of the upper to lower microvia diameter), the length of glass fiber protrusion, and the damage in the inner copper layer Cu${}_{\mathrm{damage}}$ [7,8,9], as illustrated in Figure 1. For the generation of microvias, firstly, the uppermost copper layer and then, secondly, the FR-4 is removed down to the inner copper layer using several laser pulses. After laser drilling, copper layers are affiliated by electroplating with copper to create electrically conductive connections. If voids are generated during the electroplating, the fatigue life can be significantly reduced by more than 90% [10,11]. For avoiding the generation of voids and to ensure a reliable interconnection with a high yield between outer and inner copper layers, the aims in laser microvia drilling of FR-4 PCB material are:

- Small glass fiber protrusions;
- Small taper;
- Minimal damage of the inner copper layers;
- Small variation of the microvia diameters.

A small variation of the microvia diameters with a high roundness as well as a minimal and uniform damage in the copper inner layer improves the fluid flow characteristics and thus assists the metallization process of microvias. Furthermore, smooth and uniformly sloped sidewalls with small glass fiber protrusions and tapers are easier to metallize, resulting in a higher yield [7].

Limited by the wavelength of the CO${}_{2}$ laser of λ = 9.2–10.6 µm, microvia diameters are restricted to about 40 µm [12]. In turn, by using nanosecond pulsed lasers with emission in the ultraviolet (UV) spectral region of λ = 193–355 nm, minimum hole diameters of <25 µm are achieved [3,13,14,15]. In addition, in this spectral range high absorption rates are reached for ablation of individual PCB materials such as copper, resin, and glass fiber composite material [16]. Excimer UV lasers are capable of achieving state-of-the-art microvia diameters of 20 µm [17] but are also unfavorable due to their high costs [18] and maintenance of UV optics as well as limited power availability, and additionally require enhanced safety precautions due to the use of both fluorine and chlorine.

An ultrashort pulse (USP) laser emits laser pulses in the pico- and femtosecond range [19,20] and is suitable for high-throughput fabrication of micro holes due to high attainable ablation rates and ablation efficiencies [21,22]. The short laser pulse durations lead to so-called cold ablation processes [23,24], which enable a reduction of thermal effects in laser material processing. Due to small focal diameters and negligible heat input, the drilling quality can be significantly improved [25,26,27]. In addition, processing with USP lasers is less dependent on linear absorption properties of different materials, since non-linear absorption mechanisms enable the material removal due to very high pulse peak intensities in the order of 10${}^{13}$ W/cm${}^{2}$ [28,29]. Even in materials that are usually transparent for the wavelength of the laser, an efficient absorption of light by transferring the valence band electrons to the conduction band is achievable through non-linear processes such as multiphoton or tunnel ionization [30]. USP laser beam sources with average powers of several 100 W are nowadays available [31,32], in principle allowing an increase of productivity in microvia drilling. The ablation rate is associated to the average power of a laser system (defined by the laser pulse energy and the laser pulse repetition rate [33]) and is currently not inhibited by technology. Rather, the use of higher laser pulse energies and laser pulse repetition rates is limited by occurring pulse to pulse interaction processes and the deflection speed of the applied laser scanning system, respectively [33,34,35].

If laser pulse repetition rates of several hundred kHz are used for percussion drilling of microvias, pulse to pulse interactions must be considered, which have a strong influence on the precision, productivity, and heat impact in laser material processing [25,36,37,38]. The interaction processes are, in general, distinguished into athermal and thermal processes. An athermal interaction occurs when a laser pulse interacts with laser-induced plasma or particles induced by a previous laser pulse. As a consequence, incident laser light can be absorbed, scattered, or reflected, leading to a shielding effect on the surface of the workpiece [39,40,41]. If the time between two consecutive laser pulses is not sufficient to dissipate the induced heat into the surrounding volume, thermal interaction processes occur. In this case, the workpiece is heated up by successive laser pulses, leading to heat accumulation effects [42].

For ultrashort pulsed laser drilling of metals applying laser pulse durations of 0.8–19 ps, Ancona et al. [25,36] show that the effects of particle shielding above 200 kHz is responsible for a significant increase in the required number of laser pulses to create a through hole. In addition, although the heat accumulation is suitable for shortening the processing time and thus optimizing the process efficiency, negative influences on drilling quality occur due to the formation of melt. Those findings have also been confirmed by Finger et al. [42] and Kononenko et al. [43] using picosecond laser pulses for the investigations of laser percussion drilling of steel and Ti-based alloy, respectively.

Studying percussion drilling of silicon, Gruner et al. [44] achieved superior process efficiency and drilling quality by using a laser pulse repetition rate of 40 MHz. Heat accumulation was identified as a significant factor enabling efficient material removal and a reduction of the processing time, allowing an effective drilling rate of up to 1000 holes per second. In turn, Döring et al. [45] show that ablated particles within a hole lead to shielding effects that reduce the process efficiency in percussion drilling of silicon. Similar to the results of Ancona et al. [25,36], heat accumulation countervails shielding effects for a laser pulse repetition rate of a few hundred kilohertz. For laser drilling of fused silica, Karimelahi et al. [46] report that an increase of the laser pulse repetition rate in the range of 200–1000 kHz leads to less uniform and increasingly damaged drilling channels with a large heat affected zone (HAZ), located outside the laser focus zone.

Opposite to these fundamental studies on the influence of pulse to pulse interactions in percussion drilling of metals, dielectrics, and glasses, the athermal and thermal interaction processes during the processing of multi-layer materials, such as FR-4 printed circuit board material, have not yet been adequately investigated. In this study, we therefore analyze the effects of pulse to pulse interactions on percussion drilling quality of microvias in FR-4 PCB material using different laser pulse durations in the pico- and femtosecond range, laser pulse repetition rates up to 400 kHz, and laser pulse energies in a range of *E* = 15.8–45.8 µJ. For evaluation of the microvia quality, the generated taper, the extension of glass fiber protrusions and damage of inner lying copper layers of microvias were determined by materialography.

## 2. Materials and Methods

### 2.1. Experimental Setup

**Figure 2:**
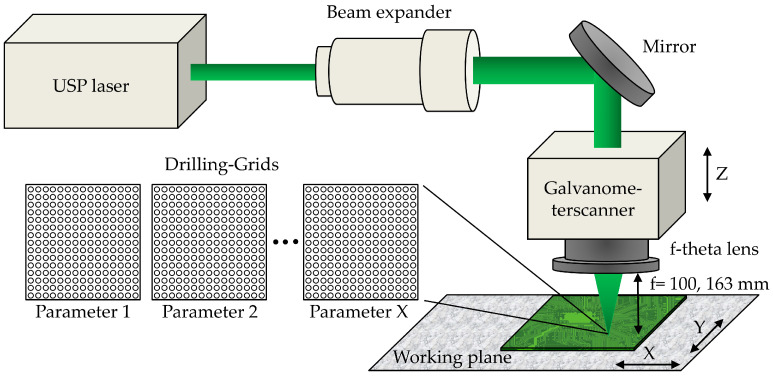
Setup for investigations of pulse to pulse interactions in the percussion drilling of microvias in FR-4 PCB material.

Experimental investigations of laser percussion drilling of PCB materials were performed with a setup including an USP laser beam source (Amphos200, Amphos GmbH, Herzogenrath, Germany; Monaco, Coherent, Inc., Santa Clara, CA, USA; Atarium XTR, Soliton Laser- und Messtechnik GmbH, Gilching, Germany), a beam expander (S6EXZ series, Sill Optics GmbH & Co. KG, Wendelstein, Germany), and a 2D galvanometer scanner (RTA-AR-800-3G, Newson NV, Dendermonde, Belgium). The simplified optical setup is illustrated in Figure 2. A galvanometer scanner equipped with telecentric f-theta lenses having focal lengths of *f* = 100 and 163 mm, respectively, were used for deflection and focusing of the laser beam, which was enlarged by a beam expander for reducing the laser focal diameter. Three different USP laser with emission in the green spectral range of λ = 515–517 nm were employed in the experimental studies, owing to different requirements for laser pulse repetition rate, laser pulse energy, laser fluence, and laser pulse duration (specifications listed in Table 1).

For the characterization of the ablation behavior of individual PCB material using different laser pulse durations of τ = 0.9 and 6 ps and laser fluences of up to 11.5 J/cm${}^{2}$, laser system 1 was used with a focal spot diameter of 30 µm. Using a laser focal spot diameter of 28 µm, laser system 2 was applied for determining the influence of different laser pulse durations of τ = 0.23, 1 and 2 ps on microvia percussion drilling quality. Laser system 3 was used to analyze the microvia drilling quality at different laser pulse energies of *E* = 15.8–45.8 µJ in combination with laser pulse repetition rates up to 400 kHz using a focal spot diameter of 26 µm.

An overview of the different laser beam configurations for the experimental investigations of ultrashort pulsed laser drilling of microvias is pointed out in Table 1. For the determination of the laser focal diameter (1/e${}^{2}$), first, the raw beam diameter (1/e${}^{2}$) was measured by a CMOS camera (IDS uEye UI-1490SE) mounted prior to the 2D galvanometer scanner. Afterwards, the laser focal diameter was calculated by substituting the measured raw beam diameter and the experimental data in Equation (1), where *f* is the focal length, λ is the wavelength of the laser, $M^{2}$ defines the beam quality, and $d_{0}$ is the raw beam diameter.

(1) $$d_{f}=\frac{4\cdot f\cdot \lambda \cdot M^{2}}{d_{0}\cdot \pi }.$$

### 2.2. Microvia Characterization

**Figure 3:**
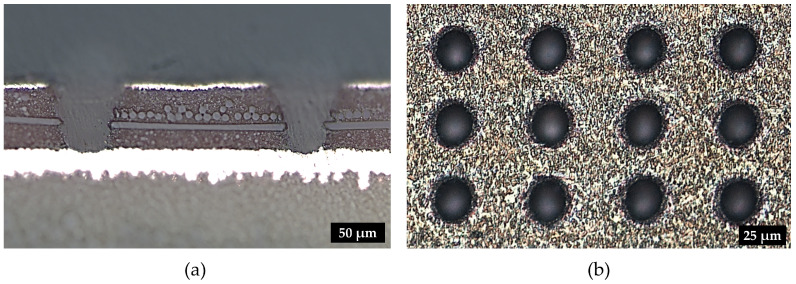
Evaluation of the microvia quality after laser percussion drilling process using (**a**) materialography and (**b**) optical microscopy.

In this study, top and bottom diameter D_top,bottom_, the length of glass fiber protrusion and the damage of the inner copper layer Cu${}_{\mathrm{damage}}$ were inspected by materialography, as depicted in Figure 1 and Figure 3a. For the determination of the microvia diameters, top view images were evaluated using optical microscopy, cf. Figure 3b. The taper is defined by the ratio of the microvia top to the bottom diameter, whereby a reference taper of 125% is used for the evaluation of the microvia drilling results. This taper is between classes A and B of taper specification for microvias [47] and is suitable for High Density Interconnect (HDI) PCBs. The glass fiber protrusion lengths are specified with a maximum of 10% with respect to the microvia diameter, since larger protrusions increase the void formation during electroplating process with copper [8].

### 2.3. Materialographic Preparation and PCB Material

For the determination of the ablation thresholds of individual PCB materials, micro holes were drilled into pure copper and FR-4 material using a number of laser pulses between 15–30 (FR-4) and 30–60 (copper), laser pulse durations of τ = 0.9 and 6 ps, and laser fluences between *F* = 1.3–11.5 J/cm${}^{2}$. Furthermore, for analyzing the influences of pulse to pulse interactions in microvia percussion drilling, microvia drilling grids were generated in FR-4 PCB material as depicted in Figure 2 using different laser pulse durations of τ = 0.23, 1 and 2 ps, laser pulse repetition rates of *f* = 5–400 kHz, laser pulse energies of *E* = 15.8–45.8 µJ, and laser fluences between *F* = 1.4 and 4 J/cm${}^{2}$.

In order to evaluate the quality of laser drilled microvias, the individual drilling grids were laser cut out of the FR-4 PCB material. After laser cutting process, first, top view images were generated using an optical microscope (Leica DM6000 M) to determine the diameter of the microvias, cf. Figure 3b. Afterwards, the cut-out samples were embedded with a 2-component synthetic material based on modified polyester resin (Demotec 15 plus) for materialographic preparation. Finally, the hardened material was removed with a grinding and polishing machine (Latzke LS3V) to examine the glass fiber protrusion length, taper, and damage in the inner copper layer in the cross section using optical microscopy, as depicted in Figure 3a.

The studied FR-4 PCB material consists of a 25 µm thick glass/epoxy composite material that separates a 5 µm and 16 µm outer and inner copper layer, respectively.

## 3. Results and Discussion

### 3.1. Ablation Thresholds

**Figure 4:**
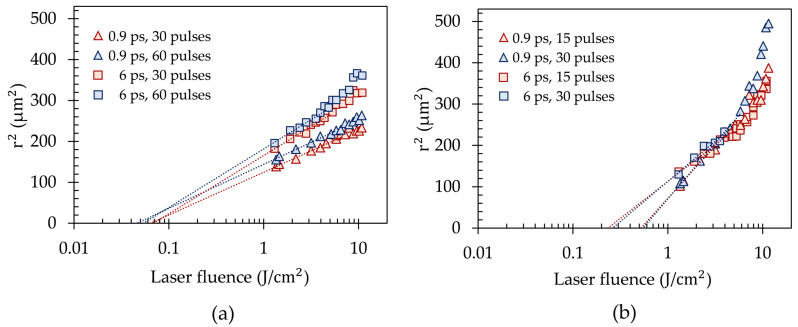
Ablation thresholds for (**a**) copper and (**b**) FR-4 using laser fluences of *F* = 1.3–11.5 J/cm${}^{2}$, laser pulse durations of τ = 0.9 and 6 ps, and a number of pulses between 15 and 60.

The ablation thresholds of multi-material PCB were determined by the so-called Liu method [48], which can be applied for pulsed laser radiation using a Gaussian intensity distribution for metals and dielectrics [49]. The determination of the specific material ablation threshold requires the radius of ablation *r* at different laser fluences. The laser fluence is defined by

(2) $$F=\frac{E}{A}=\frac{E}{w_{0}^{2}\cdot \pi }$$

where *E* is the laser pulse energy and $w_{0}$ the radius of the beam waist (1/e${}^{2}$). As a result of a non-circular laser beam with a roundness of 83% (defined by the ratio of the measured minimum to the maximum raw beam diameter), an elliptical shape of the laser ablations was observed. Consequently, each pattern was measured with respect to the minimum and maximum ablation diameter using optical microscopy. The average of both diameters is used for the determination of the ablation thresholds for copper and FR-4. The logarithmic representation of the squared drilling radii as a function of the laser fluence allows the determination of the ablation thresholds $F_{th}$. The results for copper and FR-4 are shown in Figure 4 for a different number of laser pulses between 15 and 60 using laser pulse durations of τ = 0.9 and 6 ps at a laser pulse repetition rate of 10 kHz.

As previously reported, two different logarithmic ablation regimes of ultrashort laser pulses at low and high laser fluences can be identified [50,51,52], which are, however, significantly more pronounced for the FR-4 composite material in Figure 4b as compared to copper in Figure 4a, for which this behavior becomes only apparent for a laser pulse duration of 6 ps at laser fluences above about *F* = 8 J/cm${}^{2}$. For the ablation thresholds determination, the data in the low fluence ablation regime were used. Whereas at low laser fluences the energy deposition is determined by the optical penetration depth, at higher laser fluences the effective heat penetration depth dominates the ablation [51,52]. Table 2 summarizes the ablation threshold results for copper and FR-4 by using different laser pulse durations in the pico- and femtosecond range and number of laser pulses.

The ablation threshold of copper is identified in a range between 0.05 J/cm${}^{2}$ and 0.07 J/cm${}^{2}$ by applying 30 and 60 laser pulses and laser pulse durations of τ = 0.9 and 6 ps, respectively. In comparison to the copper ablation threshold results of Raciukaitis et al. [53] by using the Liu method with a comparable number of laser pulses between 10 and 100, a laser pulse duration of τ = 10 ps, a wavelength of λ = 1064 nm, and a laser pulse repetition rate of *f* = 1 kHz, our determined ablation threshold area for copper is about one order of magnitude lower. This is the result of the shorter laser pulse durations and the higher absorption coefficient of copper for visible laser radiation [20,54]. A reduction of the laser pulse duration to τ = 0.9 ps does not cause a significant reduction of the ablation threshold, which is not consistent with the findings of Furusawa et al. [51], observing a reduction of the ablation threshold by decreasing the laser pulse duration in a range of τ = 0.12–0.8 ps for a central wavelength of λ = 780 nm. Please note, Furusawa et al. used a different method [27,52] to calculate the ablation threshold for applied laser fluences of *F* > 1 J/cm${}^{2}$. In addition, shorter laser pulse durations in the femtosecond range as well as a longer wavelength were applied in their experimental investigations.

The ablation threshold of FR-4 composite material is determined in a range of $F_{th}$ = 0.52–0.55 J/cm${}^{2}$ for applying 15 and 30 laser pulses and a laser pulse duration of τ = 0.9 ps, whereas for τ = 6 ps it is about $F_{th}$ = 0.23–0.26 J/cm${}^{2}$. Additionally, the ablation process of FR-4 composite material requires a significantly higher laser fluence as compared to copper due to different absorption and ablation mechanisms in metals and dielectrics [55].

In conclusion, a remarkable disparity in the ablation threshold of individual multi-layer PCB materials is observed, whereby the ablation process of copper starts at a laser fluence about 9.4 times lower as compared to FR-4. This poses challenges in high throughput microvia drilling of composite materials such as a PCB with potentially suboptimal conditions for the drilling process and a deleterious heat affected zone (HAZ). As the latter is also affected by the laser pulse duration [56], the influence of the laser pulse duration on the drilling quality is studied next.

### 3.2. Influence of the Laser Pulse Duration

In order to determine the influence of different laser pulse durations of τ = 0.23, 1 and 2 ps on microvia percussion drilling quality, the typical microvia characteristics were analyzed after laser processing, cf. Figure 1. For obtaining a similar averaged damage depth in the inner copper layer in a range of 2.3 µm to 2.6 µm using different laser pulse durations, the number of applied laser pulses were adapted to each other to 62 (0.23 ps), 52 (1 ps), and 65 (2 ps) at a laser pulse energy of *E* = 10 µJ and a laser pulse repetition rate of *f* = 50 kHz.

**Figure 5:**
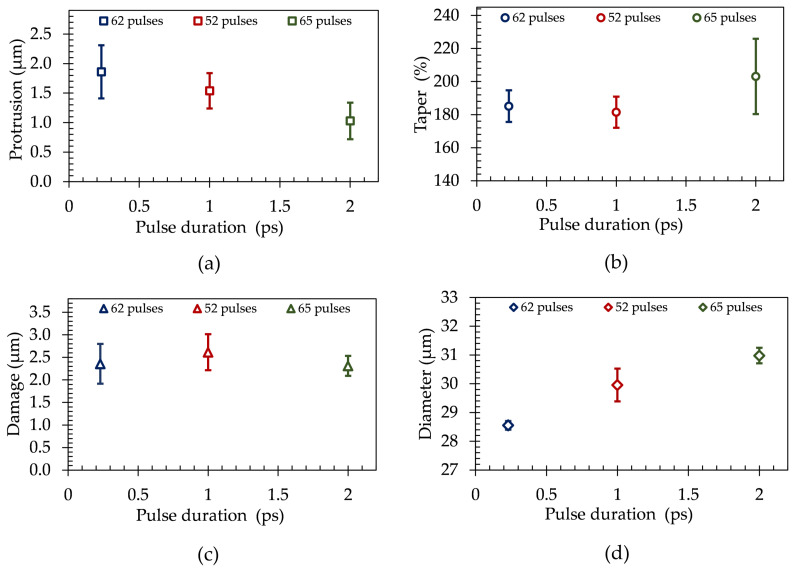
(**a**) Glass fiber protrusion, (**b**) taper, (**c**) damage in inner copper layer and (**d**) diameter of 10 laser drilled microvias in FR-4 PCB material depending on the laser pulse duration of τ = 0.23, 1 and 2 ps using a laser pulse repetition rate of *f* = 50 kHz, a laser pulse energy of *E* = 10 µJ and a number of laser pulses between 52 and 65.

For different laser pulse durations in the pico- and femtosecond range, the characteristics of 10 laser drilled microvias are illustrated in Figure 5 by their mean values and standard deviations. A comparison of the maximum glass fiber protrusion length at different laser pulse durations is depicted in Figure 5a. It is clearly visible that a longer laser pulse duration in the picosecond range is more suitable for separation of the glass fibers in the FR-4 composite material, although all investigated laser pulse durations fulfill the glass protrusion length requirements of 10% with respect to the achieved microvia diameters in Figure 5d. Using a laser pulse duration of τ = 2 ps results in an averaged glass fiber protrusion of 1 µm, which is about 45% and 33% lower compared to laser pulse durations of τ = 0.23 and 1 ps, respectively. We attribute this to a significantly higher heat input due to the longer pulse-material interaction, which causes melting of the glass fibers, resulting in smaller glass fiber protrusions.

The resulting taper are nearly constant at an averaged level of about 183.3% with a standard deviation of 9.5% by applying laser pulse durations of τ = 0.23 and 1 ps. In comparison to this, a longer laser pulse duration of 2 ps results in a 19.8% higher taper on average, cf. Figure 5b. All in all, in this laser parameter regime the achieved taper does not fulfill the requirements of taper < 125% and thus are not suitable for HDI technology in printed circuit board fabrication.

The evaluation of the damage depths in the inner lying copper layer by using different laser pulse durations of τ = 0.23, 1, and 2 ps in combination with a number of laser pulses between 52 and 65 is illustrated in Figure 5c. It is clearly visible that the required stopping process on the inner copper layer can be performed most accurately with the longest laser pulse duration of τ = 2 ps. Here, the standard deviation of 0.22 µm is about half as large as compared to the other laser pulse durations at a comparable copper damage depth. Though the thermo physical conditions leading to a more precise stopping process at the inner copper layer using a laser pulse duration of 2 ps remain complex, it can be generally assigned to a combination of a lower pulse peak intensity of the picosecond laser pulses at a constant laser pulse energy, a higher heat input due to the longer laser pulse duration as compared to femtosecond pulses, and heat dissipation in the plane of the copper layer.

In addition, the microvia diameter increases in an averaged range of 28.6 µm to 31 µm, applying a longer laser pulse duration in the picosecond range, which is shown in Figure 5d. Here, the microvia diameter increases by an average of 2.4 µm as compared to τ = 0.23 ps. Furthermore, the lowest standard deviation of the microvia diameters of 0.16 µm is achieved by using femtosecond laser pulses. In comparison to a laser pulse duration of τ = 1 and 2 ps, the standard deviations of the microvia diameters increases by 285.2% and 74.5%, respectively.

### 3.3. Influence of the Laser Pulse Energy and Repetition Rate

In ultrashort pulsed laser percussion drilling of microvias, pulse to pulse interactions must be taken into account using high laser pulse repetition rates and laser pulse energies as they can affect laser process efficiency as well as drilling quality, and thus influence the reliability and yield of microvias. For the determination of the influence of athermal and thermal interaction processes on microvia drilling quality, high laser pulse repetition rates of up to 400 kHz in combination with different laser fluences in a range of *F* = 1.4–4 J/cm${}^{2}$ were applied for microvia fabrication in FR-4 PCB material.

**Figure 6:**
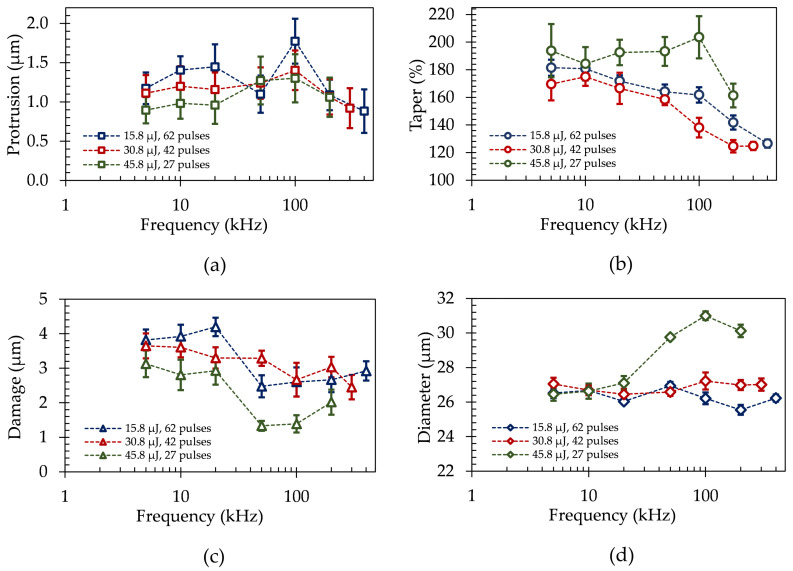
(**a**) Glass fiber protrusion, (**b**) taper, (**c**) damage in inner copper layer, and (**d**) diameter of 10 laser drilled microvias depending on the laser pulse energy of *E* = 15.8–45.8 µJ and laser pulse repetition rate of *f* = 5–400 kHz using a laser pulse duration of τ = 2 ps.

The characteristics of laser drilled microvias are shown in Figure 6 in dependence of the number of laser pulses between 27 to 62, a laser pulse repetition rate of *f* = 5–400 kHz, a laser pulse energy of *E* = 15.8, 30.8, and 45.8 µJ, and consequently a laser fluence of *F* = 1.4, 2.7 and 4 J/cm${}^{2}$. Please note, the data shown consist of the averaged values of 10 measured microvias and their standard deviations. For laser repetition rates above *f* = 200 kHz, glass fiber protrusion lengths decrease, which can be assigned to heat accumulation within the microvia, and which in turn results from the low heat diffusivity in FR-4 of about 0.002 cm${}^{2}$/s [57]. This promotes heating up the material within an area defined by the laser spot diameter over a period of about 10 µs [58], melting glass fibers and epoxy material to smooth, and cleaning the walls inside the microvias.

Heat accumulation also positively influences the occurring taper for higher laser pulse repetition rates and laser fluences of *F* = 1.4–2.7 J/cm${}^{2}$, cf. Figure 6b, reaching a level of 141.8–124.6% for laser pulse repetition rates of *f* ≥ 200 kHz by melting and broadening the bottom of the microvia. This fulfills the typical requirements for the taper geometry and is therefore suitable for HDI technology in PCB manufacturing. It is noteworthy, that for the highest applied laser fluence of *F* = 4 J/cm${}^{2}$, the taper does not reach the lowest values. This can be attributed to the significantly increased top diameter of the microvias, as shown in Figure 6d for laser pulse repetition rates of *f* > 20 kHz. This, again, is ascribed to heat accumulation due to a limited thermal conductivity out of the process zone and an increasing absorption of the copper surface for a higher laser fluence [59] and temperature [60], further reducing the ablation threshold of copper.

The depth of the inner copper layer damage, as depicted in Figure 6c, decreases with an increasing laser pulse repetition rate due to laser-induced plasma and particle shielding effects, once the inner lying copper layer is ablated inside the microvia. This can be evaluated by considering the overall energy input required to successfully drill one comparable microvia as a result of the applied number of laser pulses and the laser pulse energy. Employing a laser pulse energy of 15.8 µJ for ultrashort pulsed laser microvia drilling, a total energy input of 979.6 µJ was required, whereas for a laser pulse energy of 30.8 µJ and 45.8 µJ an overall energy input of 1293.6 µJ and 1236.6 µJ were necessary, respectively. Consequently, an increase of the required total energy for drilling one individual microvia by up to 32% is observed for laser fluences of *F* > 1.4 J/cm${}^{2}$

**Figure 7:**
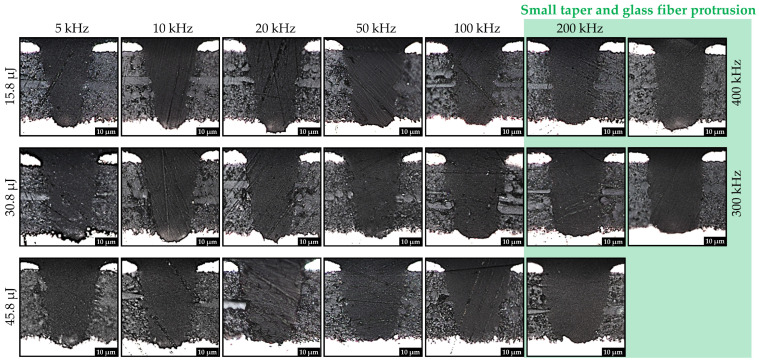
Selected cross sections of microvias in FR-4 PCB material at different laser pulse energies of *E* = 15.8–45.8 µJ and laser pulse repetition rates of *f* = 5–400 kHz using a laser pulse duration of τ = 2 ps, observing an decrease in taper for laser pulse repetition rates of more than 100 kHz.

The significantly improved microvia percussion drilling quality, particularly smaller taper ratios and smaller glass fiber protrusion length as well as lower damage to inner lying copper layers, with increasing laser pulse repetition rate of *f* > 100 kHz, is highlighted in Figure 7 by means of selected materialographic samples of microvias at laser pulse repetition rates of *f* = 5–400 kHz and laser pulse energies of *E* = 15.8–45.8 µJ using optical microscopy.

## 4. Conclusions

We have reported on the influence of the laser pulse duration, laser pulse repetition rate, laser fluence, and laser pulse number on the quality of microvia drilling in FR-4 printed circuit board material using ultrashort pulsed lasers with emission in the green spectral range. Particular focus is attended on the optimization of taper and the reduction of glass fiber protrusions and damage of inner lying copper layers, respectively. The results are discussed in respect of the different ablation thresholds of copper and dielectric compound material, heat accumulation, and shielding effects for higher laser pulse repetitions rates exceeding *f* = 200 kHz. As an overall result, using a laser pulse duration of 2 ps for microvia percussion drilling reveals superior quality with respect to minimized glass fiber protrusions, whereas applying laser pulse repetition rates of *f* ≥ 200 kHz in combination with laser fluences in a range of *F* = 1.4–2.7 J/cm${}^{2}$ lead to a minimum taper of 124.6%, fulfilling typical requirements for HDI technology in PCB manufacturing.

## Figures and Tables

**Table 1:** Technical specification of the ultrashort pulse laser systems and experimental data of the used laser beam configurations for experimental investigations of microvia drilling.

Laser Sources:	Laser System 1 (Amphos 200)	Laser System 2 (Coherent Monaco)	Laser System 3 (Atarium XTR)
Technical specifications:			
Wavelength λ (nm)	1030/515	517	1030/515/343
Laser pulse duration τ (ps)	0.9–10	0.23–5	<2
Beam quality $M^{2}$	<1.5	<1.2	<1.3
Max. laser power *P* (W)	200	30	15
Max. laser pulse energy *E* (μJ)	1000	40	300
Max. repetition rate *f* (MHz)	40	0.75	0.5
Experimental data:			
Raw beam diameter $d_{0}$ (mm)	5.3	2.8	3.3
Focal length of the f-theta lens (mm)	163	100	100
Laser focal diameter $d_{f}$ (μm)	30	28	26
Subject of investigation:	Determination of the ablation thresholds of copper and FR-4	Influence of different laser pulse durations on microvia quality	Influence of laser pulse energy and repetition rate on microvia quality

**Table 2:** Ablation threshold results for FR-4 composite material and copper at different laser pulse durations and number of applied laser pulses.

Material	Laser Pulse Duration (ps)	Number of Laser Pulses	Ablation Threshold (J/cm${}^{2}$)
FR-4 composite material	0.9	15	0.52
		30	0.55
	6	15	0.23
		30	0.26
Copper	0.9	30	0.06
		60	0.07
	6	30	0.05
		60	0.05
